# Clinical nursing effect of comprehensive nursing in department of respiratory medicine

**DOI:** 10.3389/fmed.2025.1601061

**Published:** 2025-06-04

**Authors:** Caihong Shen, Min Zhang, Ting Yao, Hui Sun, Yantian Nie, Pengfei Song, Yuan Cheng, Lu Niu

**Affiliations:** ^1^Department of Respiratory and Critical Care Medicine, The First People’s Hospital of Lianyungang, Lianyungang, China; ^2^The First Affiliated Hospital of Kangda College of Nanjing Medical University, Nanjing, China

**Keywords:** department of respiratory medicine, comprehensive nursing, clinical effect, quality of care, nursing efficiency

## Abstract

**Objective:**

To probe the clinical effect of comprehensive nursing in department of respiratory medicine.

**Methods:**

A total of 176 patients who were diagnosed and treated in the department of respiratory medicine from January 2020 to June 2022 were chosen. Based on the random lottery method, there were 90 cases in the observation group (OG) and 86 cases in the control group (CG). Patients in the CG adopted routine nursing services, while patients in the OG adopted comprehensive nursing program. The blood gas related index levels, nursing satisfaction, quality of life scores, nursing quality, patients’ anxiety, depression and adverse events were compared between the two groups after nursing intervention.

**Results:**

The nursing efficiency of the OG was increased compared to the CG. The levels of PaO2 and PaCO2 in the OG were elevated relative to the CG (*p* < 0.05). The satisfaction of patients in the OG was enhanced in contrast to the CG (*p* < 0.05). The incidence rate was 2.22% in the OG and 8.48% in the CG. The nursing quality score of the OG was elevated in comparison with the CG (*p* < 0.05). The scores of anxiety and depression in the OG were increased compared to the CG (*p* < 0.05).

**Conclusion:**

The application of comprehensive nursing in the clinical nursing process of respiratory medicine department can effectively improve the patient’s nursing satisfaction, reduce the incidence of adverse events, improve the quality of nursing, and control the patient’s negative emotions, so that the effect of patients in clinical treatment can be improved and can be vigorously promoted.

## 1 Introduction

The increasing frequency of respiratory diseases is caused by population aging and environmental pollution, and the number of patients is increasing year by year, which increases the burden of diagnosis and treatment (1, 2). The condition of respiratory diseases is relatively serious and prolonged, and elderly patients are more likely to be accompanied by complications, which affect their daily life (3, 4). The main disease types are asthma, respiratory tract infection and tuberculosis, and the treatment cycle is long, so it is necessary to receive correct nursing while standardized treatment (5, 6). Conventional nursing care pays more attention to the outcome of the disease, and the nursing form is single, lack of analysis and improvement of the nursing status, and has obvious limitations (7). Comprehensive nursing is a comprehensive and spiraling nursing program, which requires several cycles of existing nursing methods and improvement after finding problems, which is more in line with clinical needs (8). Its nursing goal is to optimize the service level, improve the quality of nursing, maximize the avoidance of nurse–patient disputes, and is widely used by multi-departments (9). This study was to probe the clinical nursing effect of comprehensive nursing in department of respiratory medicine.

## 2 Clinical data and methods

### 2.1 General clinical data

A total of 176 patients who were diagnosed and treated in the department of respiratory medicine from January 2020 to June 2022 were selected. All patients were diagnosed with respiratory diseases by arterial blood gas analysis including pH, partial pressures of arterial carbon dioxide (PaCO_2_) (PaCO_2_) and partial pressures of oxygen (PaO_2_) using Roche cobasb123 blood gas analyzer (Roche USA) and chest computed tomography (CT) using GE Optima CT660 64-slice spiral CT scanner (General Electric Company, USA), met the treatment standards of the department, and gave fully informed consent to this study. The study was approved by the Ethics committee of our hospital. Patients with mental disorders, abnormal liver and kidney function, and malignant tumors were excluded. Patients were divided into a control group (CG, *n* = 86) and an observation group (OG, *n* = 90) based on the different nursing methods.

### 2.2 Methods

Patients in the CG were given routine nursing service, behavior nursing and drug nursing. The nursing staff in the CG should adhere to the principle of “people-oriented,” and pay attention to the implementation process of nursing should highlight the humanized nursing management. Nursing staff explained to patients about the comfortable position of bed rest, scientific breathing and defecation methods. Excessive traction should be avoided for the patient’s body to prevent pain. Once an emergency occurs, it was necessary to contact the doctor in time to take targeted treatment methods. Especially when patients had severe pain, analgesics could be used to relieve pain.

The patients in the OG were given quality service on the basis of routine nursing during the nursing process, and the specific content was as follows: (1) Basic nursing: first, the nursing staff comprehensively evaluated the patient’s condition and self-care ability, and gave the patient professional, humane and individualized nursing service. By regular inspection in the ward, a clean, warm and comfortable hospital environment was created for the patients with digestive and respiratory diseases, and the safety handrails in the corridors on both sides of the respiratory ward were ensured to be normal, so that the ward could always be kept dry and clean. Among them, a variety of living equipment such as hair dryer, toilet and hair washing cart could be added to the patient’s ward. According to the interests of different patients, in order to effectively relieve patients’ anxiety and tension, so that patients can maintain a comfortable psychological state during the treatment, soothing music could be played in the patient’s ward, so that the nursing effect of patients in the department of respiratory medicine can be improved; nursing staff could also give strong humanistic care to patients by posting handwritten nursing blessing cards in the ward. (2) Psychological nursing: because of the long hospitalization time of patients in the department of respiratory medicine, they needed to receive a long nursing cycle. Therefore, once patients have bad emotions such as tension, anxiety or fear of treatment during the treatment, it was necessary for nursing staff to implement targeted nursing guidance according to the actual situation of patients. After comprehensive analysis of the patients’ bad psychological causes, scientific and reasonable nursing measures were given to patients. In patients after analyzing cause of adverse psychological reasons, and then the scientific and reasonable nursing measures for patients, Nursing staff communicated with patients patiently, had a comprehensive understanding of the patient’s psychological state and physical signs, tried to meet the reasonable requirements of patients, and fully respected the personal privacy of each patient. Nursing staff could explain the relevant knowledge of the disease to patients with weak psychological ability, so that patients could have a comprehensive understanding and cognition of their disease, which could eliminate the tension and anxiety during the treatment of the disease, and could actively cooperate with doctors to participate in the treatment work. More care and care should be given to patients and their families to avoid anxiety and depression, so as to ensure the clinical treatment effect of respiratory medicine. (3) Diet nursing care: high quality nursing service was performed in the management of respiratory medicine, the nursing staff took the actual condition of the patients as the basis for the implementation of nursing measures, and set up a reasonable diet structure, instructed patients to eat fresh vegetables and fruits as much as possible, and to avoid eating some spicy and raw foods, so as to ensure that the patient had adequate intake of protein and vitamin every day. In the process of giving patients diet nursing, nursing staff should help patients to establish a healthy diet awareness, avoid the patient’s digestive system from being stimulated by unreasonable diet, so as to improve the patient’s body function, immunity and resistance. (4) Health education: nurses needed to communicate with patients patiently and actively, and explained the nursing knowledge of respiratory diseases to patients and their families in detail during the treatment. By taking the patient’s cultural background, gender and age as the important basis for the formulation of nursing implementation plans, patients could fully understand the pathogenesis of their diseases, the treatment methods that could be taken and the situation after treatment, and correctly understand the importance of comprehensive nursing. Pay attention to the cultivation of patients’ self-management awareness, and let patients to understand the safety risks in the process of treatment. Patients should be actively followed up at home and by telephone after discharge to ensure the therapeutic effect of respiratory medicine.

### 2.3 Observation index

(1) Judgment criteria of nursing effect: it was divided into remarkable nursing effect, effective nursing and ineffective nursing. Effective nursing: the condition was significantly improved and the activity ability was improved; Ineffective nursing: the patient’s condition was not changed and was still in the critical stage; Nursing effect: the patient’s disease was basically recovered, and the body activity ability was recovered significantly. Nursing efficiency determinatio*n* = (total number of patients-ineffective cases)/total number of patients ×100%.

(2) The levels of blood gas related indexes, including arterial partial pressure of oxygen (PaO_2_) and arterial partial pressure of carbon dioxide (PaCO_2_) were compared between the two groups.

(3) The nursing satisfaction of the two groups was evaluated by our hospital’s self-made nursing satisfaction scale, with a full score of 100 points, the higher the score, the better nursing satisfaction.

(4) SF-36 quality of life scale was used to evaluate the quality of life of the two groups, with a full score of 100, and the higher the score, the better quality of life.

(5) The nursing quality score of the two groups was evaluated, mainly including nursing operation, nursing communication, health education, nurse–patient relationship and detail service. The total score was 100 points, and each item was 20 points. The higher the score, the higher the quality of patient care.

(6) The anxiety and depression of patients in the two groups were scored by the self-rating Anxiety Scale and the depression self-rating scale. The judgment criteria were as follows: ≤ 7 points were considered as normal, ≥ 8 points were considered as having anxiety and depression.

(7) The occurrence of adverse events during the nursing of patients in the two groups, mainly including sputum asphyxia, infection and pressure sores, etc.

### 2.4 Statistical analysis

SPSS 21.0 was used to analyze the data. Measurement data were expressed in the form of (x ± s). Normality was checked using the Shapiro–Wilk normality test, and t test was used for comparison between the two groups. Enumeration data were expressed in the form of rate (%), *χ*^2^ test was used for comparison between the two groups. If *p* < 0.05, the difference was statistically significant.

## 3 Results

### 3.1 Baseline characteristics of patients between the two groups

As Table 1 displayed, there were no differences in baseline characteristics including gender, age and disease type of patients between the two groups (*p* > 0.05), indicating comparability.

**Table 1:** Baseline characteristics of patients between the two groups.

Items	Control group (*n* = 86)	Observation group (*n* = 90)	χ^2^/t	P
Gender			0.46	0.49
Male	54 (62.79)	52 (57.78)		
Female	32 (37.21)	38 (42.22)		
Age (years)	54.82 ± 2.34	54.58 ± 2.47	0.66	0.50
Disease type			0.34	0.95
Bronchial asthma	32 (37.21)	36 (40.00)		
Bronchiectasis with infection	20 (23.26)	18 (20.00)		
Bacterial pneumonia	20 (23.26)	22 (24.44)		
Others	14 (16.27)	14 (15.56)		

### 3.2 Comparison of the effective rate between the two groups

Compared with the CG, the nursing efficiency of the OG was better (*p* < 0.05). The data of effective rate comparison are shown in Table 2.

**Table 2:** Effective rate of patients between the two groups.

Groups	Effective	Remarkable effect	Ineffective	Effective rate
Observation group (*n* = 90)	36 (40.00)	50 (55.56)	4 (4.44)	49 (95.56)
Control group (*n* = 86)	43 (50.00)	25 (29.07)	18 (20.93)	40 (79.07)
χ^2^	—	—	—	6.734
*P*	—	—	—	0.0043

### 3.3 Comparison of blood gas related indexes between the two groups

**Figure 1:**
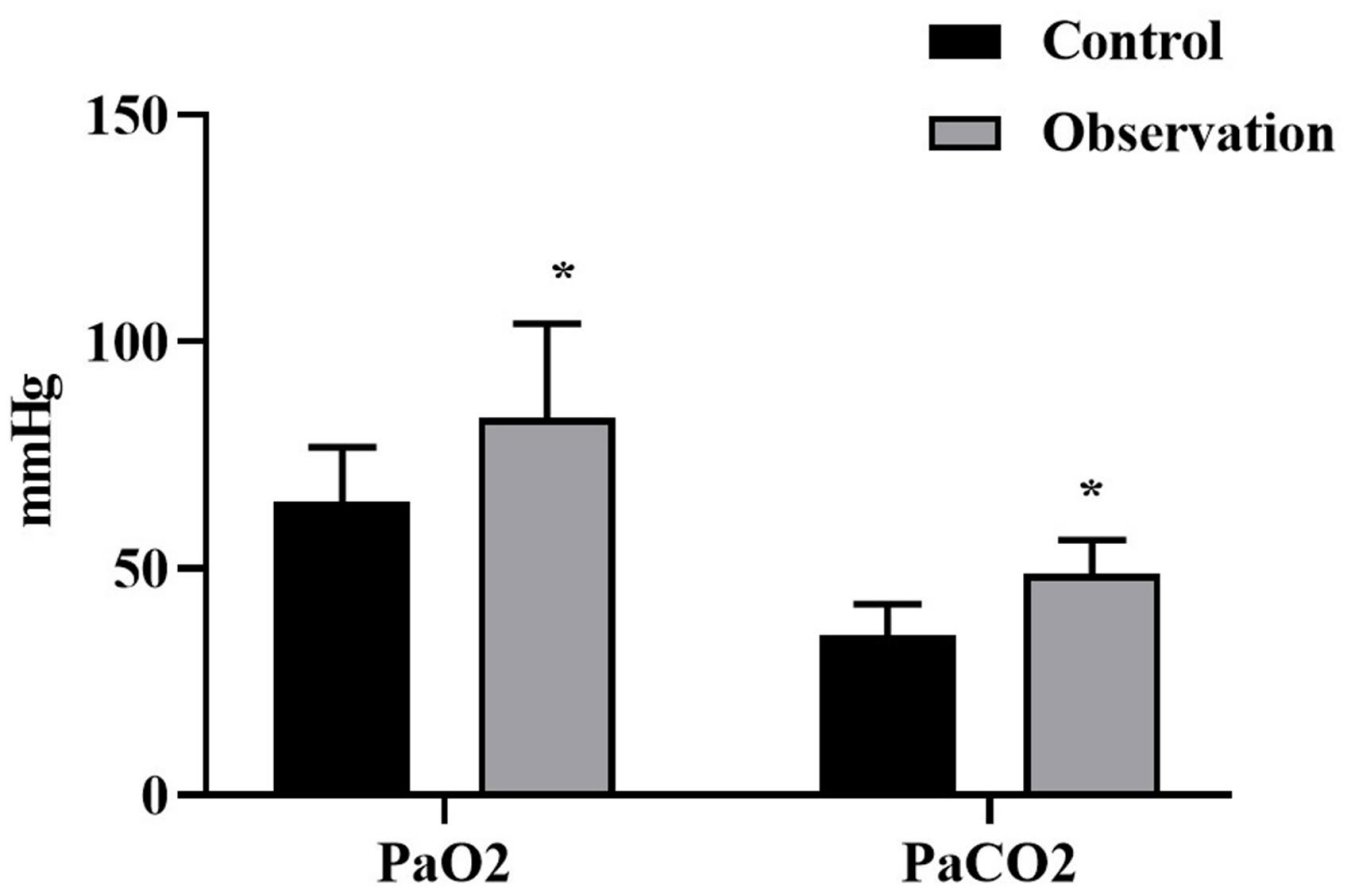
Levels of blood gas related indicators in both groups. ^*^p < 0.05, compared with control group.

The PaO2 of the OG and the CG were (83.16 ± 20.77) mm Hg and (64.54 ± 12.18) mm Hg respectively, and the PaCO2 were (35.17 ± 6.88) mm Hg and (48.68 ± 7.56) mm Hg, respectively. The levels of PaO2 and PaCO2 of the OG were better than those of the CG (*p* < 0.05), as shown in Figure 1.

### 3.4 Comparison of nursing satisfaction between the two groups

The satisfaction of the OG was increased compared to the CG (*p* < 0.05), as seen in Table 3 for details.

**Table 3:** Nursing satisfaction of the two groups of patients.

Groups	Very satisfaction	Satisfaction	Dissatisfaction	Degree of satisfaction
Observation group (*n* = 90)	57 (63.33)	33 (26.67)	0	90 (100.00)
Control group (*n* = 86)	39 (45.35)	30 (34.89)	17 (19.76)	69 (80.24)
*χ* ^2^	—	—	—	7.447
*P*	—	—	—	0.0031

### 3.5 Comparison of the incidence of adverse events during nursing between the two groups

The incidence of patients in the OG was 2.22%, and the incidence of patients in the CG was 8.48% (*p* < 0.05), as seen in Table 4 for details.

**Table 4:** Incidence of adverse events in the two groups during nursing.

Groups	Sputum asphyxia	Infection	Pressure sores	Incidence rate
Observation group (*n* = 90)	0 (0.00)	1 (1.11)	1 (1.11)	4 (2.22)
Control group (*n* = 86)	1 (1.16)	2 (2.32)	4 (4.64)	8 (8.12)
*χ* ^2^				3.614
*P*				<0.05

### 3.6 Comparison of the nursing quality scores of the two groups

**Figure 2:**
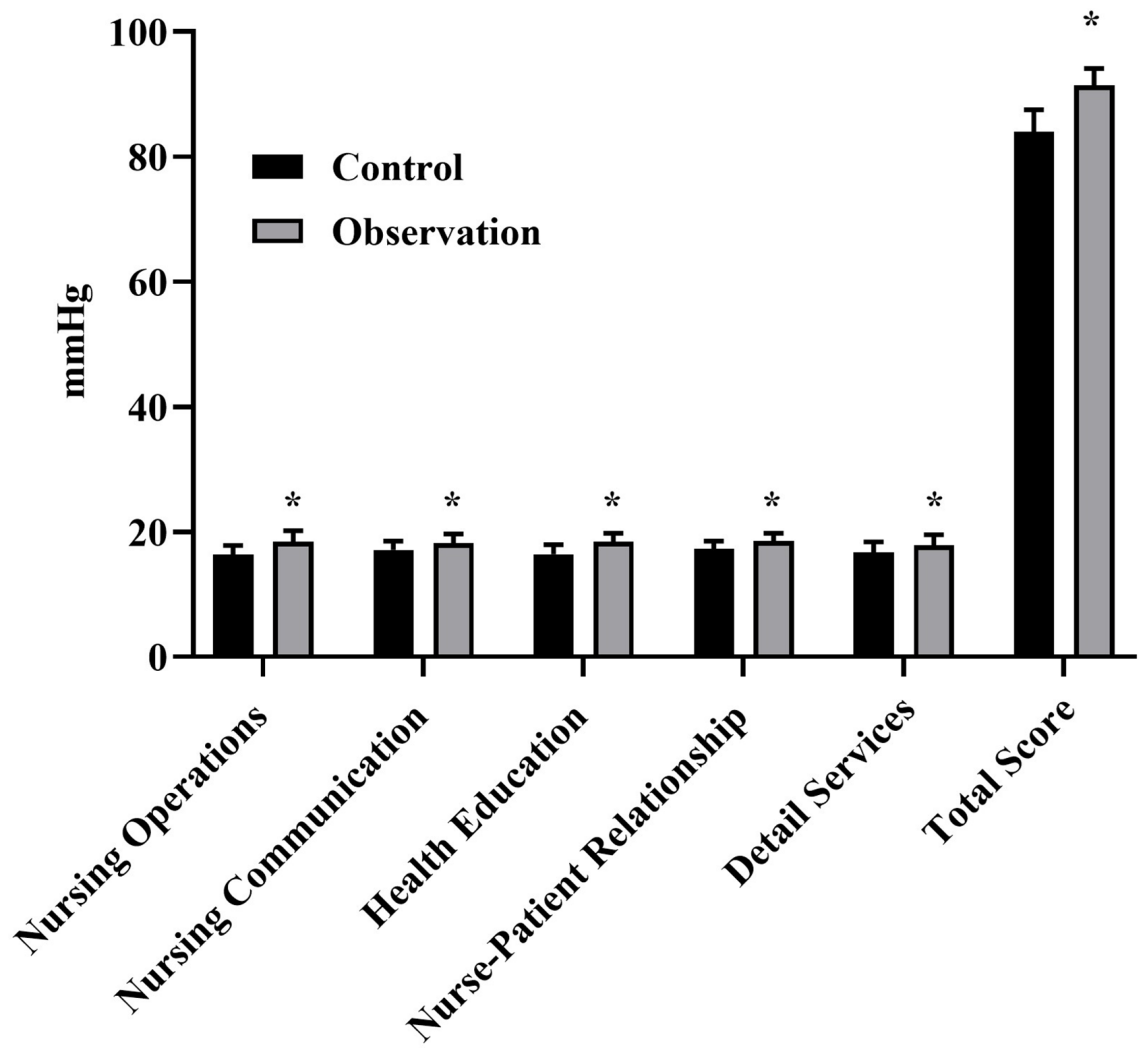
Quality of care scores for the two groups. ^*^p < 0.05, compared with control group.

The nursing quality score of the OG was elevated in comparison with the CG (*p* < 0.05), as seen in Figure 2 for details.

### 3.7 Comparison of the scores of anxiety and depression between the two groups

**Figure 3:**
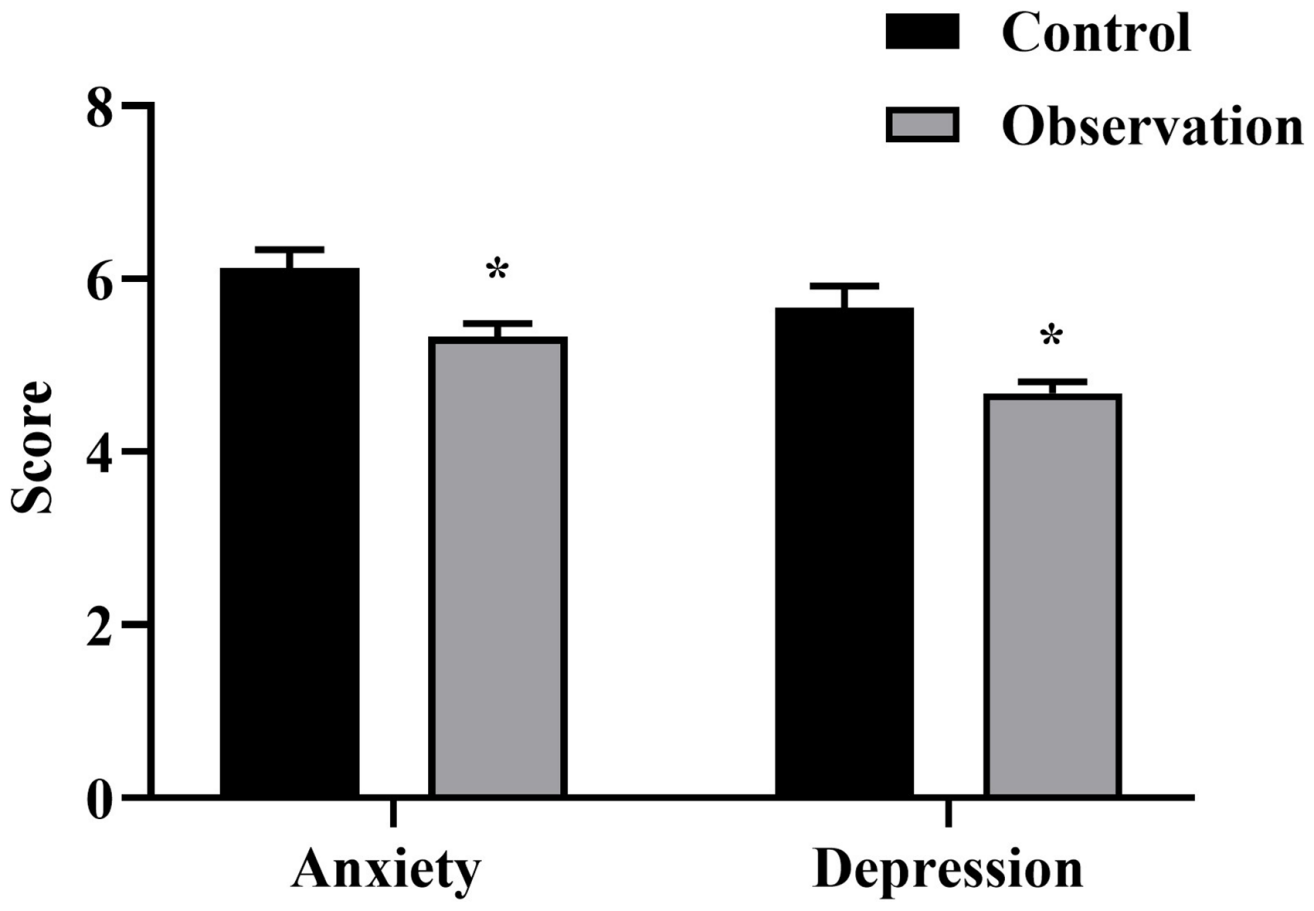
Anxiety and depression scores of the two groups. ^*^p < 0.05, compared with control group.

The scores of anxiety and depression in the OG were significantly better than those in the CG (*p* < 0.05), as seen in Figure 3 for details.

## 4 Discussion

At present, people’s requirements for medical conditions continue to improve, the traditional nursing model has been difficult to meet the development of our country’s current medical cause (10, 11). With the continuous improvement of people’s requirements for nursing quality, nursing implementation has gradually changed from “disease-centered” to “patient-centered,” with emphasis on the improvement of nursing effect (12, 13). According to research, through the applications of high quality nursing service to the clinical nursing of patients with respiratory medicine, and giving patients psychological intervention, diet nursing, health education intervention, not only can improve the treatment effect of respiratory medicine patients, but also can be effectively improve the nursing operation professional skills, safety nursing awareness and self-care ability of nursing staff, and the quality of respiratory medicine management can be effectively improved (14).

Comprehensive nursing service was adopted in the management of respiratory medicine, which involved the quality control module, basic nursing module and ward management module. Among them: (1) For the quality control module, nursing staff served as the quality control team leader, and emphasized the quality management of PDCA four stages, so as to improve the quality of nursing. By strengthening the index control of the quality control link, which specifically involved nursing factor quality indicators, link quality indicators and final quality indicators. In this way, by combining the actual situation of the whole internal medicine department to establish quality control and nosocomial infection work instructions, and use this as a basis to guide the nursing staff of internal medicine to fully realize the importance of quality control and nosocomial infection control, so that the nursing staff could be more familiar with the related nursing quality control, and truly achieve the purpose of common improvement (15). (2) Basic nursing module: nurses were required to create a clean, comfortable and quiet ward environment for the majority of patients, so that comprehensive nursing services could be truly implemented. Nursing staff should provide targeted nursing according to the patient’s condition. Especially for critically ill patients, nurses and team leaders should be responsible for the basic nursing and psychological nursing of patients to eliminate the tension and fear of patients. More humanistic care should be given to the dying patients, and psychological nursing intervention should be given when necessary, which could greatly improve the satisfaction of patients with hospital care (16). (3) Ward management module: the head nurse of the internal medicine department of the hospital was mainly responsible for drug management, material management, nursing cost accounting and patient cost management. Usually, nurses needed to classify and keep some drugs and special drugs, check the quality and validity period of drugs regularly, and then use them according to the order of validity period of drugs. Nurses needed to arrange beds for some patients who have just been admitted to the hospital, explained the health education knowledge of the disease, doctor’s advice and other policies to the patients, and avoided the situation of undercharging, overcharging and missing the patient’s expenses. The head nurse also needed to check the items regularly, formulate a scientific and reasonable item lending system, and strictly do the work of handover. Under the condition of comprehensive analysis of the workload of the internal medicine department, the number of items requested was adjusted reasonably. The responsible nurses of the department actively did a good job in the flexible exchange of idle items and nursing items from other departments to avoid serious waste. Only by truly managing consumable items, doing a good job in the maintenance of ward equipment and facilities and health management, we could provide better nursing services for patients in the department of respiratory medicine, so as to ensure the quality of nursing for patients (17).

In this paper, the nursing satisfaction of the OG was 100%, and the nursing satisfaction of the CG was 64.7%, suggesting that comprehensive nursing could effectively improve the patient’s satisfaction with nursing. Consistently, Song et al. suggested that comprehensive nursing care significantly enhanced sleep quality, speeded up rehabilitation, and increased patient satisfaction in individuals with arrhythmia after acute myocardial infarction (18).

The incidence of adverse events was 2.22% in the OG and 8.12% in the CG (*p* < 0.05), suggesting that comprehensive nursing could effectively decline the incidence of adverse events in patients with respiratory disease. In line with our finding, Xu et al. (19) suggested that comprehensive nursing was associated with the shortness of hospitalization time of patients, improved rescue rate, reduced occurrence of complications and infection, and improved satisfaction of patients.

Our study also indicated that the anxiety and depression of the OG were significantly better than those of the CG (*p* < 0.05), suggesting that comprehensive nursing could better control the patient’s negative emotions. Likewise, it has been pointed out that comprehensive physical and mental nursing for patients with acute cerebral infarction undergoing intravenous thrombolysis improves nursing efficacy, nursing satisfaction, quality of life, and patients’ psychological state (20).

In addition, the total score of nursing quality in the OG was (91.48 ± 2.63), which was better than (83.97 ± 3.52) in the CG (*p* < 0.05). This study showed that the nursing efficiency of the OG was significantly higher than that of the CG (*p* < 0.05). All these results suggested that comprehensive nursing in the clinical nursing process of respiratory medicine could effectively improve the quality of nursing.

In addition, the PaO2 and PaCO2 levels of the OG after nursing were better than those of the CG, and the quality of life score was higher than that of the CG (p < 0.05), suggesting that comprehensive nursing could effectively improve the blood gas levels and promote the quality of life in patients with respiratory disease. Consistently, Xu et al. indicated that comprehensive nursing intervention combined with respiratory functional exercises could significantly improve the pulmonary function and quality of life of patients with pulmonary tuberculosis, with obvious clinical efficacy (21).

Our research has some limitations. Firstly, the sample size of this study is relatively small and it is limited to a single center, which may reduce the universality of the research results in a broader population or different healthcare Settings. Second, the research period was relatively short and no follow-up was conducted. Therefore, more multi-center, large-scale and long-term studies need to be carried out in the future.

In summary, the application of comprehensive nursing in the clinical nursing process of respiratory medicine can effectively improve the patient’s satisfaction with nursing, decline the incidence of adverse events, improve the quality of nursing, and better control the patient’s negative emotions, so that the effect of patients in clinical treatment can be improved and can be vigorously promoted.

## Data Availability

Data generated in this study are available from the corresponding author under reasonable requests.
